# Trends in antiviral therapy of adults hospitalized with influenza in Canada since the end of the 2009 pandemic

**DOI:** 10.1186/2047-2994-3-2

**Published:** 2014-01-10

**Authors:** Geoffrey Taylor, Robyn Mitchell, Rachel Fernandes, Allison McGeer, Charles Frenette, Kathryn N Suh, Alice Wong, Kevin Katz, Krista Wilkinson, Barbara Amihod, Denise Gravel

**Affiliations:** 1 University of Alberta Hospital, Edmonton, Alberta, Canada; 2 Public Health Agency of Canada, Centre for Communicable Diseases and Infection Control, Ottawa, Ontario, Canada; 3 Mount Sinai Hospital, Toronto, Ontario, Canada; 4 McGill University Health Centre, Montreal, Quebec, Canada; 5 The Ottawa Hospital, Ottawa, Ontario, Canada; 6 Royal University Hospital, Saskatoon, Saskatchewan, Canada; 7 North York General Hospital, Toronto, Ontario, Canada; 8 Jewish General Hospital, Montreal, Quebec, Canada

## Abstract

**Background:**

Multiple observational studies have associated antiviral treatment of patients hospitalized with influenza with improved outcome, including reduced mortality. During the 2009–2010 H1N1 pandemic increased use of antiviral treatment of hospital patients was reported. We have carried out prospective surveillance for influenza in patients in a large network of Canadian hospitals since 2006. We wished to assess trends in antiviral use in the two seasons (2010–2011 and 2011–2012) since the end of the pandemic.

**Findings:**

Adults (>16 years) testing positive for influenza at the time of or during admission to participating Canadian hospitals were prospectively reviewed. In 2009–2010 there were 1132 confirmed cases, 1107 in 2010–2011 and 631 in 2011–2012. Information on antiviral therapy was available in >95% in each year. Rising to 89.6% in 2009, the proportion of adult patients treated with antiviral therapy fell to 79.9% and 65.7% in the two subsequent seasons (p < 0.001). Oseltamivir was the antiviral agent used in >98% of cases in each year. The median time from onset of symptoms to initiation of antiviral therapy was three days. The treatment proportion fell across all age groups, co-morbid conditions and disease severity.

**Conclusion:**

Despite evidence for benefit of antiviral therapy, and clinical practice guidelines recommending treatment of this population, antiviral therapy of Canadian adults hospitalized with influenza has progressively fallen in the two seasons since the end of the 2009–2010 influenza pandemic.

## Findings

### Introduction

Multiple observational studies in patients hospitalized with influenza carried out during seasonal and pandemic influenza years have documented a survival benefit in patients treated with antiviral therapy [1-4]*.* Clinical practice guidelines recommend treating all patients who are unwell enough to be admitted to hospital [5-7]*.* During the 2009 pandemic, the proportion of patients treated with antiviral therapy greatly increased in adults in US hospitals [8]*.* We have carried out prospective surveillance for the occurrence of laboratory confirmed influenza in adults admitted to a sentinel network of Canadian hospitals since 2006 and aimed to assess trends in antiviral use in this population in the two influenza seasons following the end of the 2009 pandemic.

### Setting and methods

The Canadian Nosocomial Infection Surveillance Program (CNISP) is a network of 54 largely urban tertiary acute care hospitals from ten provinces and is a partnership between the Public Health Agency of Canada which provides funding and the Canadian Hospital Epidemiology Committee, a sub-committee of the Association of Medical Microbiology and Infectious Disease -Canada. We have carried out surveillance for influenza in adult patients in network hospitals since 2006, as previously described [9]*.* Briefly, from 2006 to 2008, CNISP conducted surveillance of laboratory-confirmed influenza among hospitalized inpatients 16 years of age and older during the traditional influenza season. Following the emergence of the pH1N1 influenza in 2009, the program was expanded to year-round surveillance, which continued in the post pandemic influenza seasons. An influenza case was defined as any adult (≥ 16 years of age) with a positive influenza laboratory test result from a specimen collected during the surveillance period on or during admission to a participating hospital. Cases were identified by concurrent or retrospective chart review by infection control practitioners. Patient questionnaires were completed for each case. Patients were reviewed 30 days after initial positive test to determine whether death had occurred. Underlying medical conditions that were consistently collected for the 2009–10, 2010–11 and 2011–12 seasons were: chronic lung disease, chronic heart disease, immune suppression, diabetes mellitus and kidney disease. Descriptive statistics were calculated. Differences were assessed for categorical variables using the Chi-squared test and *p* values reflect a two-tailed alpha level of 0.05. Univariate and multivariate logistic regression and survival analysis were conducted for the data from the 2009–2010 surveillance year data onwards to assess the association between antiviral therapy use and 30-day in-hospital mortality and overall mortality respectively. In order to control for possible confounding, variables for underlying chronic lung disease, chronic heart disease and kidney disease, as well as age, were included in the final logistic and survival analysis models. Missing data and unable to assess responses were removed from all calculations. Statistical analysis was performed using Stata version 11 (StataCorp, College Station, TX) and SAS Enterprise Guide 5.1 (SAS Institute Inc, Cary, NC).

## Results

**Figure 1:**
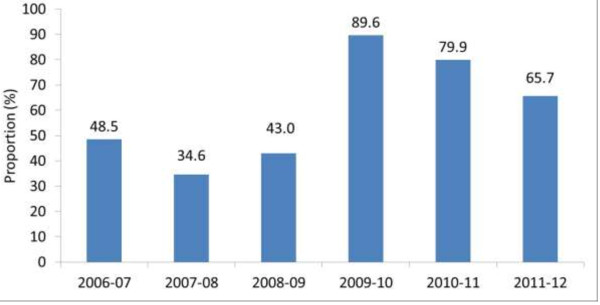
Proportion of adult inpatients with laboratory confirmed influenza who received antiviral therapy by year.

In 2009, there were 1,132 cases of influenza in 43 hospitals; antiviral therapy data was available for 1,113 cases (98.3%). In 2010–11 there were 1,107 cases in 35 hospitals; antiviral therapy data was available for 1,092 cases (98.6%). In 2011–12 there were 631 cases in 42 hospitals with antiviral therapy information available for 604 cases (95.7%). Oseltamivir was the antiviral agent used in >98% of all cases in the 3 seasons. The median duration from onset of symptoms to the start of antiviral therapy was 3 days in each of the seasons. Figure 1 demonstrates the trend in antiviral therapy in the hospital network from 2006–2012. After a marked increase in proportion of patients receiving antivirals in the 2009–2010 pandemic year compared to 2006–2007 to 2008–2009 pre-pandemic seasons, a significant and progressive decline in antiviral use occurred in the two subsequent seasons, reaching 65.7% in 2011–2012. With a few exceptions, the fall in antiviral therapy was generalized amongst participating hospitals (data not shown). Table 1 describes antiviral use in patient subgroups. In the 2011–2012 season, there was a significant decline in antiviral use in patients >65 years of age compared to 2009–2010 season (from 82.0% to 64.3%). There was a similar decline in treatment proportion in every other age category: from 91% to 63% in the 50–64 year age group, 91% to 71% in the 25–49 year age group, and 90% to 72% in the 16–24 year age group. Significant, and in some cases progressive declines in antiviral use were seen in patients with a variety of co-morbid conditions. For patients with any co-morbid condition, antiviral use fell from 89.7% in 2009 to 80.7% in 2010–2011 and 66.8% in 2011–2012. For patients ill enough to require ICU admission, antiviral therapy fell from a peak of 94.2% in 278 patients in 2009 to 79.5% of 78 patients in 2011–2012 (p < 0.001).

**Table 1:** Antiviral therapy of adults hospitalized with influenza by age, comorbidity, severity and year

**Patient subgroup**	**2009-2010**	**2010-2011**	**2011-2012**
Chronic heart disease	138 (88.5%)	315 (81.4%)	97 (66.4%)
		p = 0.045	p < 0.001
Chronic lung disease	364 (89.9%)	277 (82.9%)	123 (61.8%)
		p = 0.006	p < 0.001
Chronic renal disease	65 (89.0%)	103 (83.7%)	27 (77.1%)
		p = 0.305	p = 0.010
			
Immune suppressed	173 (91.5%)	147 (81.2%)	100 (80.0%)
		p = 0.004	p = 0.003
Diabetes	153 (92.6%)	196 (82.4%)	80 (57.6%)
		p = 0.003	p < 0.001
Pregnant	60 (88.2%)	28 (75.7%)	17 (73.9%)
		p = 0.10	p = 0.1.
Age ≥ 65 years	123 (82.0%)	607 (82.0%)	227 (64.3%)
		p = 0.994	p = 0.001
ICU admission	262 (94.2%)	120 (87.6%)	62 (79.5%)
		(p = 0.02)	(p < 0.001)

In the three seasons from 2009–2010 to 2011–2012 there were 123 in hospital deaths where a date of death was known (3.7% of all cases); 98 (79.6% of all deaths) received antiviral therapy. There was no difference in antiviral therapy among patients who died within 30 days of diagnosis across all three seasons (data not shown). Neither the univariate nor multivariate logistic regression models demonstrated a significant association between 30-day mortality and antiviral therapy use against influenza between the 2009–2010 and 2011–2012 surveillance years (data not shown). The survival analysis indicated similar results. After controlling for the effects of age, chronic cardiac disease, chronic lung disease and renal disease, antiviral therapy use was not significantly associated with the likelihood of in-hospital death. An increase in age, however, is significantly associated with an increased risk of death (see Table 2).

**Table 2:** Association between mortality and antiviral therapy, age, chronic heart disease, chronic lung disease and kidney disease

**Variable**	**p-value**	HR*- (95% CI)
Antiviral therapy	0.63	1.12 (0.72, 1.74)
Age	<0.001	1.02 (1.01, 1.03)
No chronic heart disease (reference chronic heart disease)	0.16	1.33 (0.90, 1.97)
No chronic lung disease (reference chronic lung disease)	0.93	0.98 (0.68, 1.42)
No kidney disease (reference kidney disease)	0.07	0.65 (0.42, 1.03)

Note: *HR* = hazard ratio, *CI* = confidence interval.

## Discussion

The basis for recommending antiviral therapy of patients hospitalized with influenza is derived from multiple observational studies which have demonstrated a clinical benefit in treatment of this population, including reduced mortality. This benefit has been documented for seasonal and pandemic influenza, and in ICU as well as non-ICU patients. Our data did not demonstrate an association between antiviral therapy and reduced risk of in hospital risk adjusted mortality in the pandemic and two subsequent influenza seasons; however the numbers of deaths in the three seasons, and numbers of patients who died who had not been treated with antiviral therapy were quite low (123 and 25 respectively).

While there is strong evidence for better clinical efficacy if antiviral therapy is initiated early, there is some evidence for clinical efficacy even when treatment is delayed up to 5 days following onset of illness; on theoretical grounds treatment may be effective in some subgroups when started even later [10]*.* Based on these data , the Infectious Diseases Society of America (IDSA), and its Canadian counterpart, the Association for Medical Microbiology and Infectious Diseases - Canada strongly recommend the use of antiviral therapy in all patients unwell enough to be hospitalized when treatment can be started early in the course of the illness, and recommend it when the diagnosis is made late in the course [6,7]*.*

In our hospital network, as in US hospitals, the proportion of cases admitted to hospital that were treated with antiviral agents greatly increased during the 2009 pandemic year. It is therefore disconcerting to find that the gains in treatment have been substantially lost in the post pandemic period. Garg et al. demonstrated a 6% decline in antiviral therapy of adults admitted to US hospitals from 82% during the pandemic, to 77% in the first post pandemic year [11]*.* Our data illustrates a more precipitate and progressive trend in patients hospitalized in Canadian hospitals, from the peak of 89.6% during the pandemic to only 65.7% in the second post pandemic year. This trend was widespread affecting all age groups, including the elderly, and all underlying co-morbid conditions. Furthermore it was also experienced by patients sick enough to require ICU admission.

Given the consensus that hospitalized patients should be treated, reasons for the declining use of antiviral therapy are not clear. The decline in enthusiasm for treatment may in part be driven by controversy around efficacy of antiviral therapy based on clinical trials data. Randomized trials of antiviral therapy, primarily oseltamivir, have undergone recent re-examination raising doubt regarding the clinical benefit of treatment [12]*.* However, these trials are not directly relevant to hospitalized patients, since they were conducted largely on otherwise healthy ambulatory patients.

The very high proportion of use of antivirals in 2009 occurred in the context of a pandemic and represented a marked increased compared with previous seasons. The subsequent decrease may represent a gradual return to the pre-pandemic healthcare worker behavior, or a perception that seasonal influenza, even in hospitalized patients, may be of lesser severity. This suggests that the effect of the intensive training and promotion that were conducted in the context of the pandemic was short-lived.

## Conclusion

In the two seasons following the 2009 pandemic, the proportion of patients hospitalized with influenza treated with antiviral therapy in Canada has markedly declined. Further research should be carried out to understand the reasons for declining treatment rates. Promotion of antiviral use in hospitalized patients may need to be repeated in order to obtain a sustained effect and to prevent further decrease in use.

## Competing interests

The author’s declare that they have no competing interests.

## Authors’ contributions

GT led the project, participated in protocol design and prepared the manuscript; RM participated in protocol design, analyzed the data and reviewed the manuscript. RF performed logistic regression and survival analysis. All other authors participated in protocol design and data collection reviewed the data analysis and reviewed the manuscript. All authors read and approved the final manuscript.
